# Effects of Orally Administered Resveratrol on TNF, IL-1β, Leukocyte Phagocytic Activity and Oxidative Burst Function in Horses: A Prospective, Randomized, Double-Blinded, Placebo-Controlled Study

**DOI:** 10.3390/ijms21041453

**Published:** 2020-02-20

**Authors:** Lynn M. Martin, Philip J. Johnson, Juliana R. Amorim, Amy E. DeClue

**Affiliations:** Department of Veterinary Medicine and Surgery, College of Veterinary Medicine, University of Missouri, 900 East Campus Drive, Columbia, MO 65211, USA; johnsonpj@missouri.edu (P.J.J.); jucovet@yahoo.com.br (J.R.A.); decluea@missouri.edu (A.E.D.)

**Keywords:** cytokine, endotoxin, equine, inflammation, lipopolysaccharide, nutraceutical, resveratrol, sepsis, supplement

## Abstract

Resveratrol, a phytophenol, is a commonly used equine nutraceutical supplement touted to exert anti-inflammatory effects. The effect of orally administered resveratrol on tumor necrosis factor (TNF), interleukin-1β (IL-1β), leukocyte phagocytic activity or oxidative burst function have not been reported in horses. The objective of this study was to determine the effects of a commercially available, orally administered resveratrol product on innate immune functions in healthy adult horses. Whole blood was collected from 12 horses prior to and following 3 weeks of treatment with either the manufacturer’s recommended dose of resveratrol or placebo. Phagocytosis, oxidative burst and pathogen associated molecular pattern (PAMP) motif-stimulated leukocyte production of TNF and IL-1β were compared pre- and post-treatment between treatment groups. Phagocytosis and oxidative burst capacity were evaluated via flow cytometry. Tumor necrosis factor and IL-1β were measured using cytotoxicity and ELISA assays, respectively. There were no significant differences in phagocytosis, oxidative burst or stimulated TNF or IL-1β production between resveratrol and placebo treatment groups. Orally administered resveratrol at a routinely recommended dose for a duration of 3 weeks did not significantly affect phagocytic activity, oxidative burst function or PAMP-stimulated leukocyte cytokine production.

## 1. Introduction

Long-term management of inflammation and pain in equine patients is challenging due to the detrimental side effects of prolonged drug administration. Major adverse effects of nonselective nonsteroidal anti-inflammatory drugs (NSAIDs) include gastrointestinal ulceration and nephrotoxicity [1,2]. It is well known that targeting the cyclo-oxygenase 1 (COX1) constituent isoform can result in the aforementioned side effects. Cyclo-oxygenase 2 (COX2) is the inducible isoform and therefore, COX2 selective NSAIDs have been purported to result in fewer side effects [1]. However, COX2 is also constitutively expressed in renal tissues of rats, mice, rabbits, monkeys, dogs and humans and in equine glandular gastric and bladder mucosae [3,4]. Therefore, long-term use, inappropriately high doses or increased frequency of administration could potentially result in similar side effects to nonselective NSAIDs. Opioids such as tramadol represent another long-term management option, but the oral bioavailability of tramadol is low in horses and adverse effects include muscle tremors, unsteadiness and agitation [5,6]. Upon consideration of the fact that routinely-available anti-inflammatory and analgesic drugs cannot safely be used on a long-term basis in horses, alternative therapies are needed.

Resveratrol (trans-3,5,4′-trihydroxystilbene) is a polyphenolic phytoalexin nutraceutical with pleiotropic activities [7,8]. Treatment with resveratrol has been reported to improve clinical outcomes in human patients affected with cardiovascular diseases, cancer, diabetes, obesity, metabolic syndrome, hypertension, stroke, neurodegeneration, diseases related to aging and inflammation in numerous preclinical and clinical studies [9,10,11,12,13,14]. Specific anti-inflammatory effects of resveratrol were demonstrated in clinical trials that investigated its effect during the treatment of ulcerative colitis, non-alcoholic fatty liver disease, diabetes mellitus, chronic obstructive pulmonary disease and cardiovascular disease [15,16,17,18,19]. Some studies have suggested that potent anti-inflammatory actions of resveratrol are explained by its ability to reduce cytokines including tumor necrosis factor (TNF)-α and C-reactive protein [16,20,21]. Suppression of the oxidative stress and inflammatory transcription factors Nrf2 and NF-κB, respectively, represent other reported explanations for anti-inflammatory action of resveratrol [15,22]. These demonstrated anti-inflammatory effects of resveratrol suggest that it might be useful as an adjunct to NSAID use in horses requiring long-term anti-inflammatory treatment for chronic disease.

Although the use of resveratrol as a nutraceutical with numerous purported but unproven benefits for equine health has been gaining acceptance in recent years, published studies reporting anti-inflammatory or analgesic effects in horses are few [23,24,25,26,27,28,29]. In one study of performance horses affected with distal tarsitis, oral resveratrol supplementation used in conjunction with an intra-articular corticosteroid resulted in reduced lameness when compared to intra-articular corticosteroid and an orally administered placebo [28]. Orally administered resveratrol did not affect basal circulating plasma TNF-α concentrations after being administered to lean and obese mares in another study [27]. Conversely, a reduction in basal serum TNF-α concentrations was reported following treatment of horses affected with Equine Metabolic Syndrome [30,31] with resveratrol [23]. Modulation of important antioxidant biomarkers, including reductions in malondialdehyde concentration and activity of glutathione peroxidase and increased superoxide dismutase and catalase activities, was demonstrated following the oral administration of resveratrol to horses affected with age-related osteoarthritis [24].

It is logical that, prior to recommending resveratrol as an anti-inflammatory nutraceutical for horses, evidence for an effect on innate inflammatory functions should be sought. Therefore, the objective of this study was to perform a prospective, randomized, blinded trial to investigate the effects of a commercially available, orally administered resveratrol product on stimulated TNF and interleukin-1β (IL-1β) production, leukocyte phagocytic activity and oxidative burst function in healthy adult horses. Commercially available resveratrol, orally administered at the manufacturer’s recommended dose for 3 weeks, maintained phagocytic activity and oxidative burst function and did not significantly affect pathogen associated molecular pattern (PAMP)-stimulated leukocyte cytokine production in healthy adult horses.

## 2. Results

### 2.1. Patient Population

Breeds represented were American Saddlebreds (*n* = 5), American Quarter Horses (*n* = 5), American Paint Horse (*n* = 1) and Standardbred (*n* = 1). Age ranged from 5 to 15 years (median 8 years), with all horses being geldings (castrated males). The mean leukocyte count for all horses at the outset of the study was 7363/µL (range 6270–8870; rr 5400–14,300), the neutrophil count was 4223/µL (range 3170–5490; rr 2260–8850), the monocyte count was 129/µL (range 0–410; rr 0–100), and the lymphocyte count was 2897/µL (range 1610–4350; rr 1500–7700). These results were all consistent with a healthy state. No adverse effects of resveratrol or placebo were observed.

### 2.2. Leukocyte Cytokine Production

**Figure 1:**
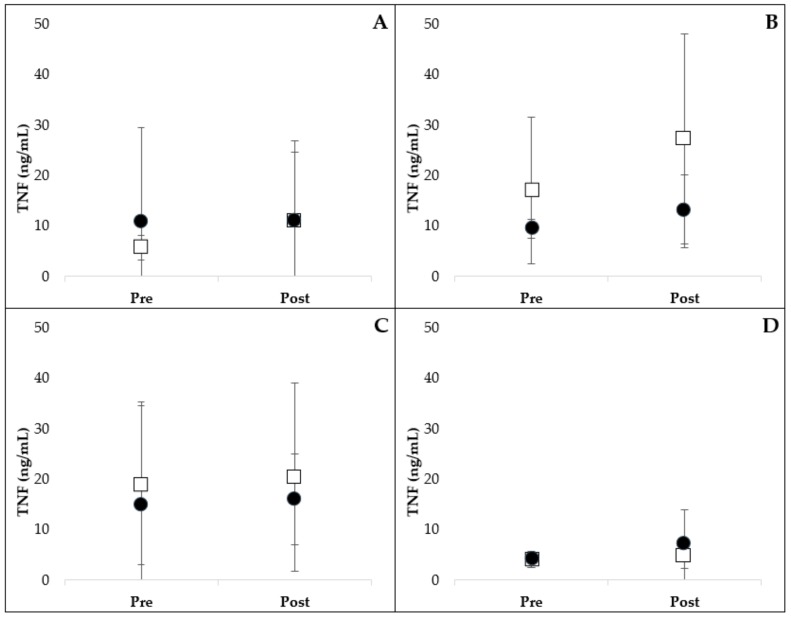
Leukocyte production of TNF following stimulation with LPS (**A**), LTA (**B**), PG (**C**) and control PBS (**D**). Pre- and post- 3-week oral administration of placebo (open square) or resveratrol (closed circle) supplement are represented. There was no significant difference in TNF production between treatment groups over time (mean ± SD). LPS, lipopolysaccharide; LTA, lipoteichoic acid; PBS, phosphate-buffered saline; PG, peptidoglycan.

The results of PAMP-stimulated leukocyte TNF production are presented in Figure 1. Extreme outlier data were removed from analysis for lipopolysaccharide (LPS)- (*n* = 1, placebo group), lipoteichoic acid (LTA)- (*n* = 1, resveratrol group) and phosphate-buffered saline (PBS)- (*n* = 1, placebo group) stimulated TNF data. Adding these data back into the analysis did not change the significance of the results. Administration of resveratrol did not alter LPS- (*p* = 0.536), LTA- (*p* = 0.290), peptidoglycan (PG)- (*p* = 0.964) or PBS- (*p* = 0.532) stimulated TNF production compared to placebo.

**Figure 2:**
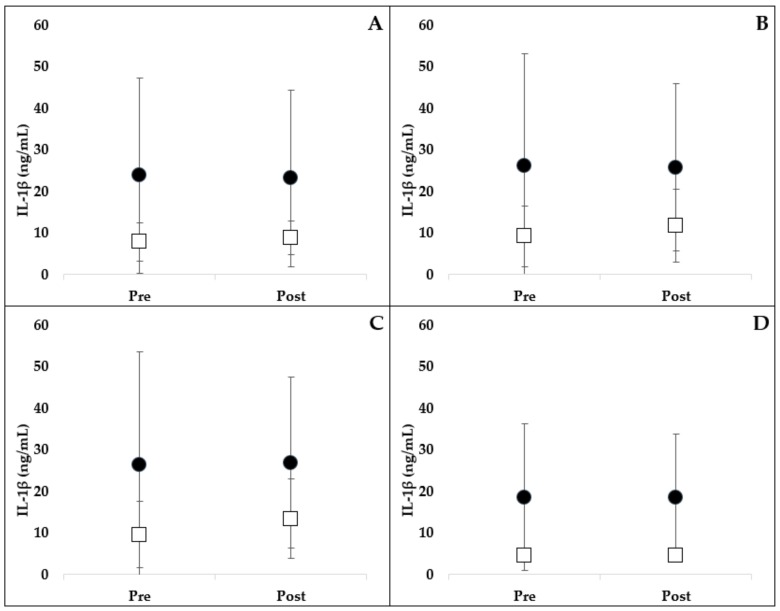
Leukocyte production of IL-1β following stimulation with LPS (**A**), LTA (**B**), PG (**C**) and control PBS (**D**). Pre- and post- 3-week oral administration of placebo (open square) or resveratrol (closed circle) supplement are represented. There was no significant difference in IL-1β production between treatment groups over time (mean ± SD). LPS, lipopolysaccharide; LTA, lipoteichoic acid; PBS, phosphate-buffered saline; PG, peptidoglycan.

The results of PAMP-stimulated leukocyte IL-1β production are presented in Figure 2. One horse (resveratrol group) was an extreme outlier in the IL-1β data, and therefore, was removed. Adding this horse back into the analysis did not change the significance of the results. Administration of resveratrol did not alter LPS- (*p* = 0.306), LTA- (*p* = 0.375), PG- (*p* = 0.347) or PBS- (*p* = 0.933) stimulated TNF production compared to placebo.

### 2.3. Phagocytosis and Oxidative Burst

**Figure 3:**
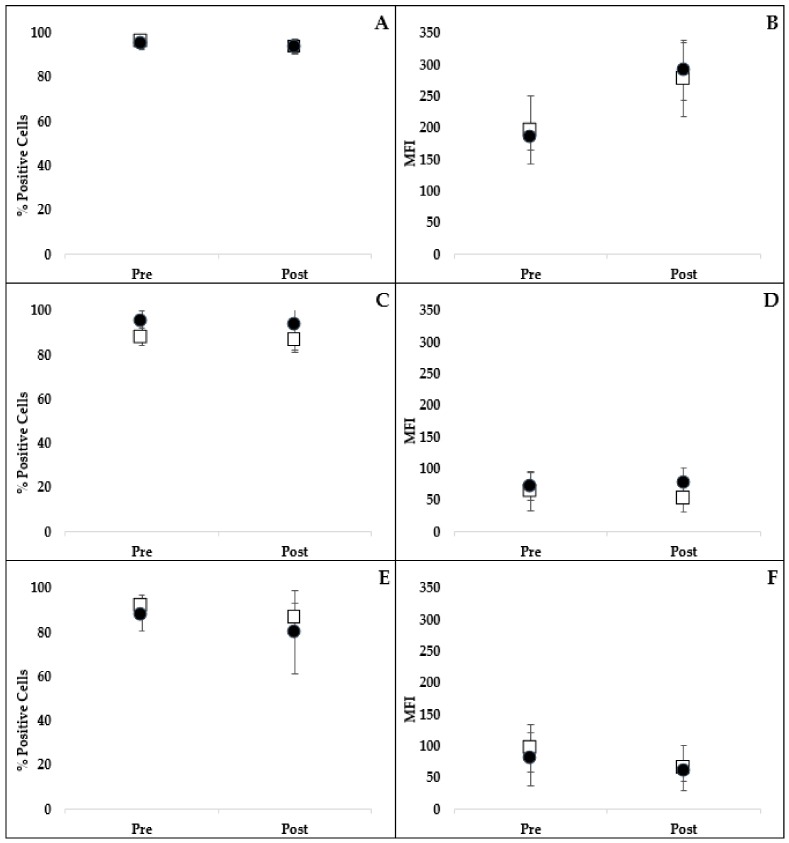
Comparison of the percentage of neutrophils and monocytes phagocytizing FITC-labeled *E. coli* (**A**) and the MFI (**B**) representing the intensity of phagocytosis following stimulation with *E. coli* pre- and post- 3-week oral administration of placebo (open squares) or resveratrol (closed circles). Comparison of the percentage of neutrophils and monocytes performing oxidative burst (**C**,**E**) and the intensity of oxidative burst (MFI; (**D**,**F**)) following stimulation with *E. coli* or PMA, respectively. There were no significant differences in phagocytosis or oxidative burst between treatment groups over time (mean ± SD). *E. coli*, *Escherichia coli*; MFI, mean fluorescent intensity; PMA, phorbol myristate acetate.

There was no significant difference in the percentage of cells performing phagocytosis of opsonized *Escherichia coli* (*E. coli)* between treatments over time (Figure 3) (*p* = 0.296). Nor was there a difference in the number of bacteria phagocytized (mean fluorescence intensity; MFI) between treatment groups (*p* = 0.445) (Figure 3).

Oxidative burst capacity of leukocytes after opsonized *E. coli* or phorbol myristate acetate (PMA) stimulation was determined using flow cytometry. There was no significant difference in the percentage of cells undergoing oxidative burst between treatments over time for either *E. coli*- (*p* = 0.658) or PMA-stimulated oxidative burst (*p* = 0.786) (Figure 3). There was no difference detected in the intensity of the oxidative burst response (MFI) after *E. coli* (*p* = 0.119) or PMA (*p* = 0.464) stimulation between treatment groups (Figure 3).

## 3. Discussion

Based on the results of this study, orally administered resveratrol at the recommended dose did not have an effect on the measured outcomes of innate immune function in healthy adult horses. We found no significant difference in PAMP-stimulated cytokine (TNF or IL-1β) production or polymorphonuclear leukocytes (PMN) phagocytosis or oxidative burst function between treatment groups. Based on routine monitoring previously described, there were no observed adverse effects in any of the subjects.

These results are consistent with our previous in vitro study in which there was no effect of resveratrol on PAMP-stimulated TNF production or PMN phagocytic or oxidative burst capacity at the tested concentrations, raising questions about in vivo efficacy in the horse [32]. Alternatively, Siard et al. (2016) evaluated the in vitro effects of resveratrol in aged horses (≥ 20 years) using a peripheral blood mononuclear cell model [33]. In that study, resveratrol performed similarly to or more effectively than phenylbutazone and/or flunixin meglumine in reducing lymphocyte derived interferon gamma and TNF-α [33]. Canine in vivo work indicated that short-term oral resveratrol supplementation resulted in increased phagocytosis by PMNs, blunted oxidative burst capacity, increased PAMP-stimulated TNF and IL-6 production and no effect on IL-10 [34]. It is argued that the preserved effects of PMN phagocytosis and oxidative burst functions represent a desirable outcome in our study as those functions are a critical first line host defense to eliminate pathogens and combat infections [35].

It is possible that the dose of resveratrol administered to horses in this study (450 mg twice daily) was not sufficient to exert an immunomodulatory effect on the measured parameters. All other in vivo equine studies employed a different commercially available resveratrol supplement, and the total dose per day was somewhat higher compared to the manufacturer’s recommendations for the supplement used in our study [23,24,25,26,27,28,29]. The formulation of the resveratrol supplement might be a contributing factor to effect (or lack thereof) because of the poor solubility and bioavailability of resveratrol, creating a major challenge for the pharmaceutical industry [36].

To the authors’ knowledge, there have been no published safety, pharmacokinetic or pharmacodynamic studies regarding the use of resveratrol in horses, and this establishes a gap in understanding of this popular phytophenol. In humans, intestinal absorption occurs rapidly following intake but the overall oral bioavailability is less than 1% [37]. Low oral bioavailability has been attributed to substantial cytochrome p450 and gastrointestinal microbiota metabolism of resveratrol [38,39]. In light of the fact that horses are ‘hindgut fermenters’ and have an extensive gastrointestinal system with distinctive populations of microbiota throughout the gastric, small intestinal and large intestinal compartments, it is plausible that the absorption and/or bioavailability of resveratrol in horses is different than humans [40]. Canine peak plasma concentration of resveratrol occurred 1–5 h after oral administration and rapid metabolism resulted in conjugation to glucuronide and sulfate metabolites [41]. Comparatively, there seems to be more extensive conjugation in humans while data for horses is lacking [42].

To underscore the need for pharmacokinetic, pharmacodynamic and safety investigations of resveratrol, a number of adverse side effects have been reported in other species following administration of high doses of resveratrol for an extended period. These side effects include gastrointestinal disturbances (nausea, vomiting, diarrhea), nephrotoxicity, hepatotoxicity and even premature death [43,44,45]. The equine supplement industry is not overseen or regulated by a licensing authority that might serve to ensure product claims, and therefore, commercial products might contain inappropriately high (or low) concentrations of purportedly active ingredients. Eighty-four percent of U.S. horse owners routinely administer nutritional supplements to their horses for purported veterinary-related issues, and the risk of over-supplementation is greater when multiple supplements are administered concurrently [46]. Additionally, respondents identified veterinarians as the primary resource for supplement information [46]. Although resveratrol toxicity seems highly unlikely due to low bioavailability reported in other species (<1% in humans, 1.5% in rabbits, 30% in rats) [37,47,48], the aforementioned side effects might be due to poorly understood resveratrol metabolites.

There was a considerable degree of inter-horse variation regarding baseline cytokine production (especially IL-1β) in cultivated whole blood. Based on previous work from our laboratory, we have observed substantial inter-horse variation in blood cytokine production in response to PAMPs. In human medicine, marked variations in cytokine production have also been identified. Elucidation of the underlying molecular mechanisms that influence inter-individual responses are deserving of further investigation in the equine species. Genetic polymorphisms have been identified as a major contributor to the degree of inflammatory response in people [49,50,51]. There is evidence that a genetic role may be important in this regard in horses as well [52]. It is also suspected that unidentified genetic polymorphisms might be an important contributing factor to inter-individual variances in our study.

Numerous other factors that may influence measured cytokine responses, including age, sex, seasonality, microbiome, socioeconomic status, percentage of body fat, drugs and physical activity level, have been reported [53,54,55,56,57,58]. Management (housing, diet and exercise) of the horses used for this study was unchanged throughout the study period. Moreover, the entire investigative period was concluded during a single season; as a species with marked seasonal influences on physiology, it is important to acknowledge that results of an investigation such as this one might yield different results at different times of the year [59,60].

All horses were deemed healthy at the outset of the study and thus, the aforementioned factors likely did not significantly contribute to the cytokine response differences between individuals in this study. Similar to what has been described in the human medical context [50,51], horses in this study fell into relatively distinct groups of high and low baseline cytokine production. For this study, horses were randomly recruited into treatment groups, resulting in a greater number of “high responders” (high baseline cytokine production; inter-horse variation) in the resveratrol treatment group than in the placebo group. Our data analysis evaluated the effect of resveratrol over time using repeated measures, which helped to exclude differences in inter-individual cytokine production at baseline. In light of a relatively low study population, we did not investigate the effects of breed or age on individual cytokine production.

Whole blood culture was used in this study to more appropriately simulate the physiologic effects in the living animal compared to peripheral blood mononuclear cell (PBMC) methods (using isolated leukocytes). This is especially important in the horse because certain plasma proteins in the blood are imperative for LPS-induced activation of neutrophils [61]. Moreover, methods that employ isolated PBMC may sometimes be unrepresentative of the physiological situation as a result of distorted monocyte:lymphotyce ratios and absence of growth factors, cytokines and other cellular components normally present in circulating blood [62,63].

While differences between high and low responders is a limitation of our study, from a clinical perspective, there are no commercially available diagnostic tests to identify horses that are likely to be high versus low responders. Therefore, resveratrol is given to the general population without regard to each individual’s expected cytokine production response. In this setting of unknown individual response characteristics, we aimed to demonstrate a treatment effect; in other words, in a clinical setting, does resveratrol have an impact? Future studies could subgroup horses into high and low cytokine responder categories to investigate if there is a subpopulation of horses that are more likely to benefit from this therapy. However, using a randomly sampled group of horses from the general population of mixed high and low cytokine responders, we were not able to demonstrate any effect of resveratrol on innate immune responses. In addition, the sample population was relatively small and thus Type II error was possible. The dose of resveratrol was based on that which is commercially available and recommended by the manufacturer. However, changing the dose or duration of administration might have changed the outcome.

## 4. Materials and Methods

### 4.1. Resveratrol and Control

A commercially available equine oral resveratrol supplement (Resvantage Equine^®^, Advantage Biosciences, Inc., Newport Beach, CA, USA) (450 mg resveratrol orally twice per day for three weeks, based on the manufacturer’s recommendation) was used for this study. An oral placebo supplement (control) was produced and supplied by the manufacturer of the resveratrol supplement. The placebo supplement had an identical capsule to the commercially available product and contained similar constituents minus resveratrol (Table S1). The individuals administering the supplement and individuals analyzing the samples in the laboratory were blinded to the treatment assignment of each animal and sample.

### 4.2. Animals and Monitoring

The Institutional Animal Care and Use Committee of the University of Missouri approved this study (protocol #7334, approved May 18, 2012) and no animals were subjected to euthanasia. All animal use experiments were performed at Stephens College Equestrian Center of Columbia, Missouri. A prospective, randomized, double-blinded, placebo-controlled clinical trial was designed for twelve, healthy adult geldings aged between 5 and 15 years. Health was based on a normal medical history, results of physical examination and a complete blood count. Exclusion criteria included any medication, vaccination or nutraceutical administration in the antecedent 4 weeks. Randomly (names drawn by hat), the horses were split between the two treatment groups, resveratrol (*n* = 6) or placebo (*n* = 6). All horses received daily exercise and were housed in a similar environment. The individuals administering the treatments monitored each subject for abnormalities or side effects by recording consumption of the supplement, behavior, mentation, appetite, urination and defecation.

### 4.3. Sample Collection

Heparinized whole blood was collected aseptically via jugular venipuncture pre-treatment (Day 0) and post-treatment (Day 21). The treatments were given with daily grain feedings (morning and evening) with confirmed consumption observed for each horse at each time of administration.

### 4.4. Leukocyte Cytokine Production

Samples were processed within 2 h of collection. Whole blood was diluted 1:2 with Roswell Park Memorial Institute medium (RPMI) containing 200 U/mL of penicillin and 200 mg/mL of streptomycin (Gibco^®^, Invitrogen, Grand Island, NY, USA) and added to 24-well plates. Samples were then stimulated with LPS from *E. coli* 0127:B83 (Sigma-Aldrich^®^, St. Louis, MO, USA) (final concentration, 100 ng/mL), LTA from Streptococcus faecalis (Sigma-Aldrich^®^, St. Louis, MO, USA) (final concentration, 1000 ng/mL), PG from *Staphylococcus aureus* (Sigma-Aldrich^®^, St. Louis, MO, USA) (final concentration, 1000 ng/mL) or PBS control was added to respective wells. Solutions were mixed on a plate rocker at room temperature for 5 min and incubated at 37 °C, in 5% CO_2_ for 24 h. Upon removal from incubation, each plate was centrifuged (400 x *g* for 5 min at room temperature) and supernatants collected and stored (−80 °C) until batch analysis [64].

### 4.5. TNF Assay

Lipopolysaccharide-, LTA-, and PG-stimulated leukocyte production of TNF was evaluated in triplicate using a cytotoxicity bioassay previously developed and validated in our laboratory [32,64,65,66,67,68]. Briefly, murine (L929) fibroblasts were cultured on 96-well plates for 12 h and then supernatants added. After a 20 h incubation with minimum essential medium (Invitrogen^™^, Carlsbad, CA, USA) plus horse serum and actinomycin D3 (Sigma-Aldrich^®^, St. Louis, MO, USA), the 3-[4,5-dimethylthiazol-2-yl]-2,5,-diphenyltetrazolium bromide (Sigma-Aldrich^®^, St. Louis, MO, USA) colorimetric assay was used to quantify the number of live cells per well. Absorption was measured at 570 nm and optical density of test wells was compared to that of wells with known concentrations of recombinant equine TNF (R&D Systems^™^, Inc., Minneapolis, MN, USA) for quantification.

### 4.6. IL-1β Assay

Lipopolysaccharide-, LTA-, and PG-stimulated leukocyte production of IL-1β was evaluated in duplicate using a previously validated equine specific ELISA kit (KingFisher Biotech^®^, Inc., St. Paul, MN, USA) according to the manufacturer’s directions with some modification [69]. Samples were diluted 1:2 with assay diluent to optimize recovery based on spike and recovery experiments performed in our laboratory (mean recovery 90.5%). Absorption was measured at 450 nm, and optical density of test wells was compared to that of wells with known concentrations of equine IL-1β for quantification. The lower limit of detection was 6.25 µg/mL. The inter- and intra-assay coefficient of variation were <15% and <5%, respectively.

### 4.7. Phagocytic and Oxidative Burst Function

The phagocytic and oxidative burst function of PMNs were determined using commercially available test kits (Phagotest^®^ and Phagoburst^®^, Opragen Pharma, Heidelberg, Germany) according to the manufacturer’s protocol as previously described [32,70,71]. For phagocytic function, blood was incubated with opsonized FITC-labeled *E. coli* strain LE392 for 10 min in a 37 °C water bath; negative control samples were incubated for the same time but without bacteria. Samples were placed on ice, which stopped phagocytosis and quenching solution was used to extinguish FITC fluorescence of surface bound bacteria. Samples were washed, red blood cells were lysed and leukocytes were fixed using diethylene glycol and formaldehyde. Propidium iodide was added to stain DNA and exclude aggregation artifacts of bacteria or dead cells during flow cytometry.

For the measurement of oxidative burst, samples were incubated with control solution and opsonized *E. coli* strain LE392 or PMA for 10 min. Formation of reactive oxidants during oxidative burst was monitored by the addition of dihydrorhodamine 123 and its oxidation to rhodamine 123. The reaction was halted by the addition of fixing and lysing solution. Propidium iodide was added to stain DNA and exclude aggregation artifacts of bacteria or dead cells during flow cytometry. Samples were placed on ice and shielded from light prior to flow cytometry.

### 4.8. Flow Cytometry

**Figure 4:**
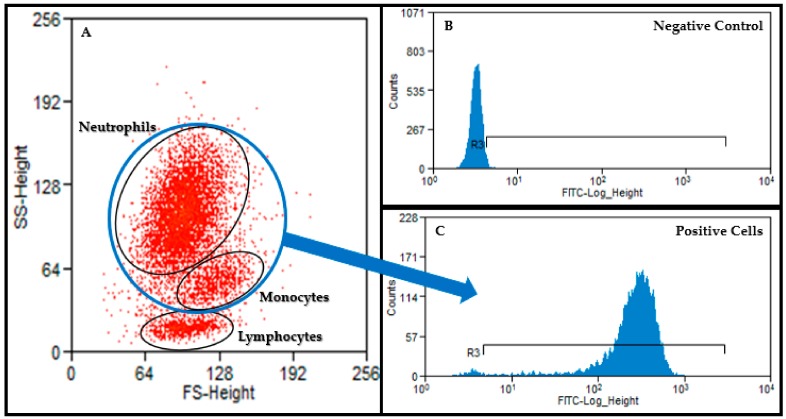
A forward versus side scatter plot (**A**) was used to gate neutrophils and monocytes. This gate was then applied to the appropriate histogram to identify FITC negative (**B**) and positive cells ((**C**); neutrophils and monocytes).

Flow cytometry was performed at the University of Missouri Cell and Immunology Core Facility using a 488 nm argon-laser (FACScan^™^, Beckman Coulter, Inc., Brea, CA, USA) and associated software (Summit V 5.2.0.7477). A minimum of 10,000 events was recorded for each sample and a forward versus side scatter plot was used for gating of leukocytes (Figure 4). Percentage of macrophages and neutrophils having performed phagocytosis as well as MFI (the number of bacteria phagocytized) were recorded for assessment of phagocytic function. The percentage of cells having produced reactive oxygen metabolites (recruitment) and the MFI (oxidative burst intensity) were recorded for assessment of oxidative burst function.

### 4.9. Data Analysis

Data were analyzed using a commercially available software (SigmaStat^®^, Systat Software, Inc., San Jose, CA, USA). Histogram plots and the Shapiro–Wilk test were used to determine whether data were normally distributed. Repeated measures ANOVA with post-hoc Tukey test or a Wilcoxon signed-rank test were used to compare differences between treatment groups over time. Extreme outliers were defined as values greater than three times the standard deviation greater than or less than the mean or more than three interquartile ranges greater than or less than the median. Statistical significance was recognized if *p* < 0.05.

## 5. Conclusions

Orally administered resveratrol at a routinely recommended clinical dose for a duration of 3 weeks did not significantly affect phagocytic activity, oxidative burst function or PAMP-stimulated leukocyte cytokine production. Further pharmacokinetic and pharmacodynamic investigations are recommended prior to recommending the routine use of commercially available resveratrol supplements for the purpose of immunomodulation in horses.

## Supplementary Materials

Supplementary Materials can be found at https://www.mdpi.com/1422-0067/21/4/1453/s1.

## Abbreviations

COX	cyclo-oxygenase
*E. coli*	*Escherichia coli*
IL	interleukin
LPS	lipopolysaccharide
LTA	lipoteichoic acid
MFI	mean fluorescence intensity
NSAID	nonsteroidal anti-inflammatory drug
PAMP	pathogen-associated molecular pattern
PBMC	peripheral blood mononuclear cell
PBS	phosphate-buffered saline
PG	peptidoglycan
PMA	phorbol myristate acetate
PMN	polymorphonuclear leukocytes
RPMI	Roswell Park Memorial Institute
TNF	tumor necrosis factor
