# Supplementary material for: Effects of Orally Administered Resveratrol on TNF, IL-1β, Leukocyte Phagocytic Activity and Oxidative Burst Function in Horses: A Prospective, Randomized, Double-Blinded, Placebo-Controlled Study
Source: Int J Mol Sci. 2020 Feb 20;21(4):1453. doi: 10.3390/ijms21041453 (PMC7073105; doi:10.3390/ijms21041453)
Supplement: Supplementary file 1 [file ijms-21-01453-s001.pdf]

Table S1: Placebo and resveratrol capsule composition

	Placebo capsule	Active resveratrol capsule
Biotin (10 mg)	●	●
Atlantic Kelp (50 mg)	●	●
Flaxseed Oil (300 mg)	●	●
Lecithin (40 mg)	●	●
Peppermint Oil (25 mg)	●	●
Resveratrol (450 mg)	○	●
